# Phylogeny and Density Dynamics of *Wolbachia* Infection of the Health Pest *Paederus fuscipes* Curtis (Coleoptera: Staphylinidae)

**DOI:** 10.3390/insects11090625

**Published:** 2020-09-11

**Authors:** Chen Ge, Jiayao Hu, Zimiao Zhao, Ary A. Hoffmann, Shuojia Ma, Li Shen, Jie Fang, Jianqing Zhu, Weidong Yu, Weibin Jiang

**Affiliations:** 1 Laboratory of Environmental Entomology, College of Life Sciences, Shanghai Normal University, Xuhui, Shanghai 200234, China; gretchen9505@163.com (C.G.); hujiayao@shnu.edu.cn (J.H.); zhaozimiao818926@163.com (Z.Z.); mashuojia@126.com (S.M.); shenlismile@163.com (L.S.); fangjie2019@163.com (J.F.); ywd@shnu.edu.cn (W.Y.); 2 School of BioSciences, The University of Melbourne, Bio21 Institute, Parkville, VIC 3052, Australia; Ary@unimelb.edu.au; 3 Shanghai Zoological Park, Changning, Shanghai 200335, China; zzzjjq@gmail.com

**Keywords:** *Paederus fuscipes*, *Wolbachia* infection, phylogeny, density dynamics

## Abstract

**Simple Summary:**

*Wolbachia pipientis* is a maternally inherited endosymbiont of arthropods and filarial nematodes, and was reported to occur in *Paederus fuscipes*, a beetle that causes dermatitis linearis and conjunctivitis in humans when they come in contact with skin. In this study, we report the phylogenetic position and density dynamics of *Wolbachia* in *P. fuscipes*. The phylogeny of *Wolbachia*, based on an analysis of MLST genotyping, showed that *Wolbachia* from *P. fuscipes* belongs to supergroup B. Quantitative PCR indicated that the infection density in adults was higher than in any other life stage (egg, larva or pupa), and that reproductive tissue in adults had the highest infection densities, with similar densities in the sexes. These findings provide a starting point for understanding *Wolbachia* infection dynamics in *P. fuscipes*, and interactions with other components of the microbiota.

**Abstract:**

The maternally inherited obligate intracellular bacteria *Wolbachia* infects the reproductive tissues of a wide range of arthropods and affects host reproduction. *Wolbachia* is a credible biocontrol agent for reducing the impact of diseases associated with arthropod vectors. *Paederus fuscipes* is a small staphylinid beetle that causes dermatitis linearis and conjunctivitis in humans when they come into contact with skin. *Wolbachia* occur in this beetle, but their relatedness to other *Wolbachia*, their infection dynamics, and their potential host effects remain unknown. In this study, we report the phylogenetic position and density dynamics of *Wolbachia* in *P. fuscipes*. The phylogeny of *Wolbachia* based on an analysis of MLST genotyping showed that the bacteria from *P. fuscipes* belong to supergroup B. Quantitative PCR indicated that the infection density in adults was higher than in any other life stage (egg, larva or pupa), and that reproductive tissue in adults had the highest infection densities, with similar densities in the sexes. These findings provide a starting point for understanding the *Wolbachia* infection dynamics in *P. fuscipes*, and interactions with other components of the microbiota.

## 1. Introduction

*Wolbachia pipientis* is the most widespread endosymbiotic bacterium of insects and other arthropods, infecting perhaps two-thirds of present-day insect species, as well as about 40% of terrestrial arthropod species [1]. The transmission of *Wolbachia* is predominantly vertical and secondarily horizontal [2]. It can induce a number of reproductive manipulations in its host, including cytoplasmic incompatibility [3], thelytokous parthenogenesis [4], feminization of genetic males [5] and male killing [6]. *Wolbachia* may generate positive fitness effects on numerous hosts, such as filarial nematodes, fruit flies, bedbugs and wasps [7,8,9,10], and decrease host transmission of dengue [11], malaria [12], West Nile virus [13] and other pathogens [14]. It is considered as a novel method for controlling mosquito- and vector-borne human diseases [15]. The vector control approaches include population suppression [16,17,18] and population replacement strategies [19]. The population suppression approaches involve rearing and releasing large numbers of male mosquitoes that cannot produce viable offspring when they mate with wild females. By contrast, population replacement approaches involve the release of both male and female mosquitoes that carry a heritable factor that reduces or blocks their ability to transmit viruses [15].

*Wolbachia* infections have been reported in various Coleoptera families, such as Buprestidae, Chrysomelidae, Curculionidae, Dytiscidae, Gyrinidae, Haliplidae, Hydraenidae, Hydrophilidae, Noteridae, Staphilinidae and Tenebrionidae, but usually only with a limited coverage of species [20,21,22,23,24,25,26]. *Paederus fuscipes* Curtis is a widespread beetle, with a distribution from the British Isles in the east, across Central Asia to Japan, and southeast to Australia. Although *P. fuscipes* preys on several agricultural pests and represents an important beneficial insect [27], it can also adversely affect human health, because its vesicant hemolymph can cause dermatitis linearis and conjunctivitis if it comes into contact with human skin [28,29,30]. *P. fuscipes* neither bite nor sting, but can cause dermatitis linearis and conjunctivitis by accidental brushing or crushing of the insects over an exposed area of the human skin. The symptoms are due to a toxic substance named pederin released from their hemolymph [31]. *P. fuscipes* was originally examined with respect to *Wolbachia* infection by Yun et al. [26], and its infection status was recently confirmed by Maleki-Ravasan et al. [24]. Yun et al. [26] found the indirect horizontal transmission of *Wolbachia* between rove beetles and their predator spiders, while Maleki-Ravasan et al. [24] provided an estimate of *Wolbachia* prevalence (76%, 95/125) in *P. fuscipes* in Iran. However, little is known about other aspects of this infection, including its tissue distribution patterns and density dynamics in *P. fuscipes*.

The tissue distribution of *Wolbachia* in its hosts is often uneven [32]. Based on initial studies in mosquitoes and *Drosophila*, high densities of *Wolbachia* were found in reproductive tissues [3,33,34], which was thought to be connected to transovarial transmission and the ability of *Wolbachia* to influence host reproduction [35]. A wider somatic tissue distribution of *Wolbachia* has been reported in other arthropods, such as isopods [36], triatomine bugs [37] and bean beetles [38]. *Wolbachia* density also varies between life stages, and can shift in density towards specific organs during development [39].

In this study, we characterized the *Wolbachia* in *P. fuscipes* by MLST genotyping. Furthermore, we measured *Wolbachia* density across all the developmental stages, body parts and tissues of *P. fuscipes* with qPCR. The *Wolbachia* spatiotemporal infection density in beetles may help to indicate the likely effects of *Wolbachia* on this host. 

## 2. Materials and Methods

### 2.1. Samples and DNA Extraction

A laboratory stock of *P. fuscipes* was established from 33 adult beetles (18 females and 15 males) collected in Nanyang, Henan province, China, in May 2019. They were fed separately under greenhouse conditions at 25 °C, 60% relative humidity and a photoperiod of 16 h of light and 8 h of darkness. To establish isofemale lines, beetle pairs were kept in a fixed order in perforated plastic boxes, as described by Kellner and Dettner [40], with some leaves for shelter, and a small dish containing moistened cotton in which to lay the eggs. The females were fed with pork liver powder and honey (50 μg for one beetle per day) and were allowed to lay eggs seven days later. The eggs were isolated for hatching, and the isofemale line was established using resulting sibling larvae.

DNA was isolated from different developmental stages of the F1 generation (egg, larva, pupa and adult) and parental samples. Nine rove beetles were tested per developmental stage, except for the eggs, which were tested in nine groups of three eggs (n = 27 in total). The tissues (gut and reproductive tissue) and body parts (head, thorax, and abdomen without the gut and gonads) were dissected from other adult beetles (9 males and 9 females). Each tissue sample was dissected from a beetle. The method of dissection was carried out following Kador et al. [41]. The DNA was isolated from the dissected body parts and tissues using a QIAamp DNA Mini kit (Qiagen, Hilden, Germany) following the manufacturer’s instructions [42]. 

### 2.2. Wolbachia Screening and Multilocus Sequence Typing

To screen for the presence of *Wolbachia*, a region of 870 bp in length was amplified from all the samples using general *Wolbachia* primers for 16S rRNA [43] (Table 1). The PCR reactions followed the published protocols [44]. The characterization of *Wolbachia* strains was performed by sequencing multiple loci recommended by the *Wolbachia* MLST database (http://pubmlst.org/Wolbachia) [45,46] (Table 1). The MLST typing included sequencing fragments from five *Wolbachia* genes: *gatB*, *coxA*, *hcpA*, *ftsZ* and *fbpA*. 

The MLST data were aligned with a homologous sequence of a wide range of arthropods retrieved from the *Wolbachia* MLST database (http://pubmlst.org/Wolbachia) as well as from the NCBI database (Supplementary Materials Table S1). These sequences were aligned with manual correction using Bioedit v. 7.0 [47]. The best-fit partitioning scheme and corresponding nucleotide substitution models for the concatenated matrix were selected by PartitionFinder v2.1.1 [48] using the Bayesian Information Criterion (BIC). The GTR+R model is the best-fit substitution model for five partitions. The concatenated supermatrix was analyzed with maximum likelihood (ML) inference using IQtree 1.4.2 [49]. IQtree is an efficient software for phylogenomic inference. A combination of hill-climbing approaches and astochastic perturbation method can be time-efficiently implemented. To assess nodal support, we performed 1000 ultrafast bootstrap replicates and an SH-aLRT test with 1000 replicates. The UFBoot is largely unbiased compared to standard or alternative bootstrap strategies, and SH-aLRT is conservative [50,51,52]. Only nodes with support values of UFBoot ≥ 80 and SH-aLRT ≥ 75 were considered robust. 

### 2.3. qPCR and Statistical Analyses

To measure the infection dynamics of *Wolbachia* across all tested developmental stages, body parts and tissues of *P. fuscipes*, qPCR was performed in triplicate for each sample using Platinum SYBR Green (Invitrogen) referring to the manufacturer’s protocol. qPCR reactions were performed in a total volume of 20 μL, comprising 10 μL of 2X Platinum SYBR Green, 0.4 μL (5 μM) of each primer and 1 μL (final 5 ng) template DNA. Following Ali et al. [53], the relative *Wolbachia* density was calculated as the ratio of Cq values between the *Wolbachia* surface protein gene (*wsp*) and the host’s ribosomal protein S3 gene (RPS3), which is synonymous with the number of *Wolbachia* per host cells, because both genes occur as a single copy per haploid genome. The short fragment length (158 bp) of the *Wolbachia* targeted primer pair (wsp1-F1-wsp1-R1) was used and normalized with a 191bp fragment length of the reference gene (RPS3-F, RPS3-R; Table 1) [53,54]. Relative expression levels were calculated using the 2^−ΔΔCt^ method [55]. The temperature profile of the qPCR was 94 °C for 4 min, 40 cycles of 95 °C for 30 s, 50 °C for 30 s, and 72 °C for 45 s with fluorescence acquisition of 72 °C at the end of each cycle, then a melting curve analysis after the final cycle. Assays were conducted as three technical replicates. 

We checked for the normality and homoscedasticity of the data prior to using parametric statistical tests. We compared *Wolbachia* infection densities among the different developmental stages, body parts and tissues of *P. fuscipes* by ANOVA followed by a multiple comparison test (Tukey’s posthoc test). We used *t*-tests to compare *Wolbachia* densities between males and females. All analyses were conducted using SPSS statistics version 21.0 for Windows (SPSS Inc, Chicago, IL, USA).

## 3. Results

**Figure 1:**
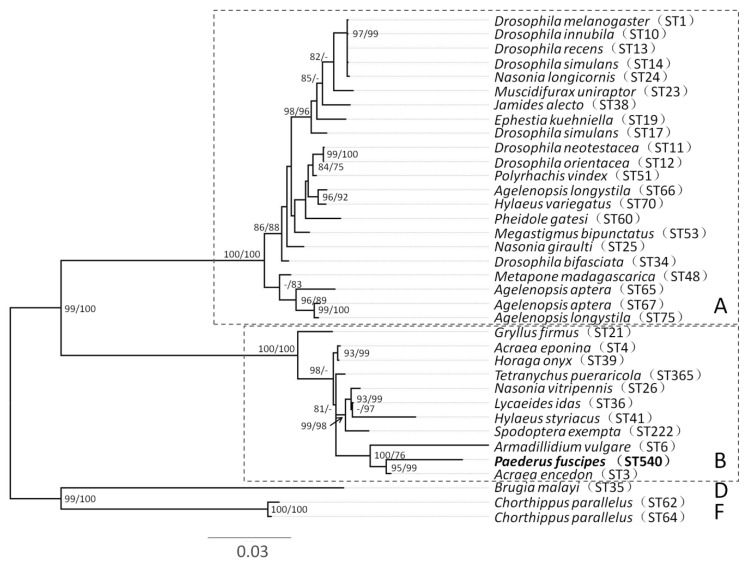
Maximum-likelihood phylogenetic tree of *Wolbachia* MLST sequences from *P. fuscipes* and additional ST sequences from a wide range of host species. The phylogeny is inferred by IQTREE. Numbers beside nodes are IQTREE ultrafast bootstrap and SH-aLRT values. The affiliation to the respective supergroup (**A**, **B**, **D**, **F**) is indicated.

All rove beetles examined by diagnostic PCR for 16SrRNA were *Wolbachia*-infected. All individuals appeared to have a single infection based on unambiguous electropherograms. The sequence typing of these individuals produced new alleles for the *hcpA* and *coxA* loci, with *ftsZ*, *fbpA* and *gatB* matching existing alleles in the database. The strain identified by the *Wolbachia* MLST database has the designation ST-540. The phylogenetic trees for concatenated alignment were constructed and showed that ST-540 belonged to supergroup B (Figure 1). The most closely related strain was a male-killing *Wolbachia* (ST-3) in the butterfly *Acraea encedon* [56]. 

**Figure 2:**
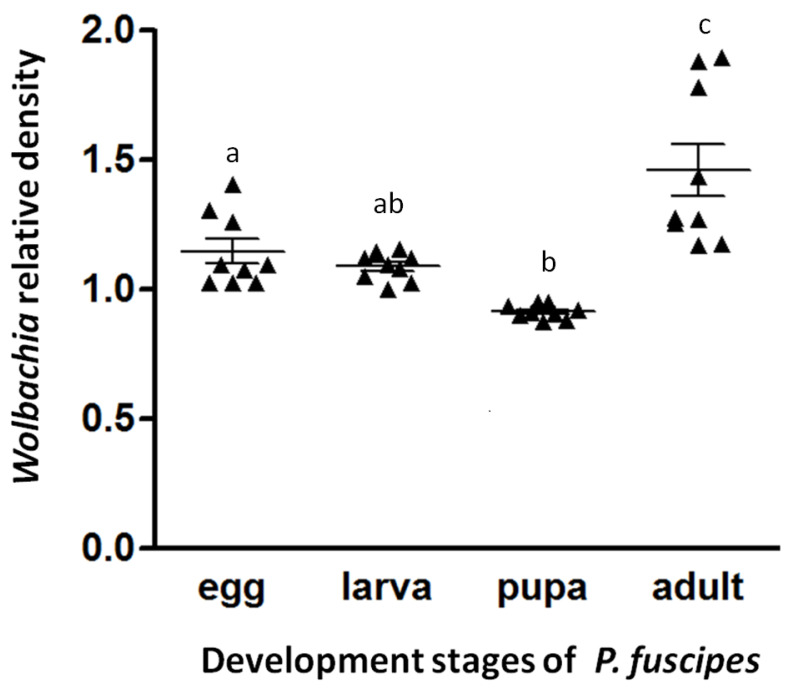
Relative *Wolbachia* density was measured across different developmental stages. Nine biological replicates were tested for each development stage. This would include both the individuals used from larvae to adults, and the egg pools. Each data point represents the average of three technical replicates. The bars represent mean ± standard error (n = 9) and the different letters above the scatter dot plot indicate a significant difference between developmental stages (*p* < 0.05).

**Figure 3:**
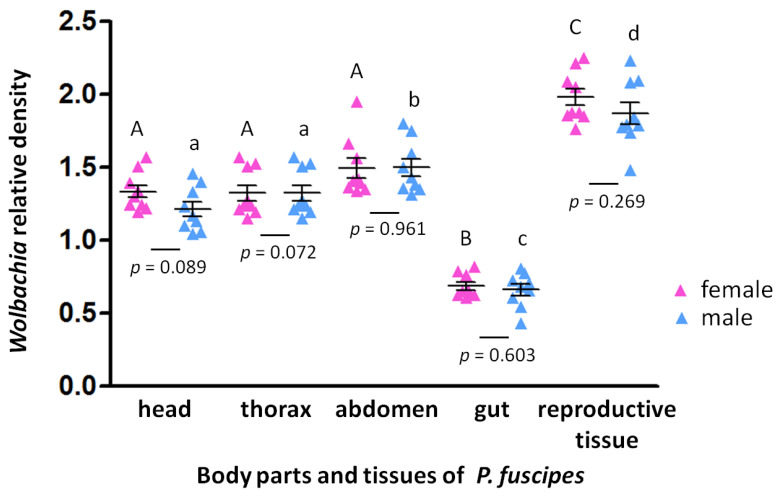
Relative *Wolbachia* density was measured across different body parts and tissues. Nine *P. fuscipes* were tested per treatment. Each data point represents the average of three technical replicates. The bars represent mean ± standard error (n = 9) and the different letters above the scatter dot plot indicate significant difference between developmental stages (*p* < 0.05). Uppercase letters represent female while male is represented by lowercase letters.

The *Wolbachia* infection densities analyzed through qPCR with the specific *wsp* gene along with an endogenous control gene (RPS3) were found to vary significantly (F_(3,32)_ = 16.023, *p* < 0.01) across the developmental stages. The infection density in adults was significantly higher than in any other life stage (Figure 2). Moreover, the *Wolbachia* infection density significantly varied between host body parts and tissues, both in females (F_(4,40)_ = 79.783, *p* < 0.01; Figure 3) and males (F_(4,40)_ = 68.353, *p* < 0.01; Figure 3), with significantly high infection densities in reproductive tissues and lower densities in the gut (Figure 3). However, the relative *Wolbachia* densities between females and males for body parts and tissues were not significantly different. The densities of *Wolbachia* are therefore substantially influenced by developmental stage and tissues, but not gender. 

## 4. Discussion

Based on phylogenetic reconstructions, *Wolbachia* species exist in 17 supergroups designated by the letters A–R, with supergroup G being controversial [57,58,59]. The *Wolbachia* infections in Coleoptera characterized so far belong to supergroups A, B or F. In total, 12% of Coleopteran species tested to date harbored *Wolbachia* from supergroup A, another 12% harbored *Wolbachia* from supergroup B and only three species harbored *Wolbachia* from supergroup F [22]. In this study, *Wolbachia* infections screened from all tested samples of *P. fuscipes* were positive and belonged to supergroup B. The *Wolbachia* from the B supergroup in Coleoptera may affect beetle hosts in several ways. They have been shown to induce cytoplasmic incompatibility in *Altica lythri* from Central Europe [21], *Callosobruchus chinensis* from Japan [60] and *Conotrachelus nenuphar* from the USA [61]. Additionally, they have been suspected as inducing parthenogenesis in *Aramigus conirostris* from South America [62], and male killing in *Adalia bipunctata* from Russia [63].

We provided a quantitative analysis of *Wolbachia* infection densities across different development stages, body parts and tissues of *P. fuscipes* by qPCR. All individuals were *Wolbachia* positive, suggesting accurate *Wolbachia* vertical transmission by a parent to its offspring. *Wolbachia* density in adults was higher than in any other life stage (eggs, larvae and pupae) while the infection density in pupae was lowest (Figure 2). While there is a statistical difference, this difference may not equate to any biological differences. The *Wolbachia* density dynamics for the life stages in *P. fuscipes* were in accordance with those for three other Coleopteran species, *Tribolium confusum* [31], *Octodonta nipae* [53], and *Brontispa longissima* [64]. *Wolbachia* may be subject to the differential control of proliferation during the development of hosts [31]. The high *Wolbachia* density in adults and in eggs may be caused by functional associations with those host tissues. Since *Wolbachia* are primarily vertically transmitted from mothers to offspring through the egg cytoplasm, *Wolbachia* density is expected to be higher in the reproductive tissues of adults and in eggs [31,53,64]. Many studies have reported that *Wolbachia* display a strong tropism for the germline so as to ensure vertical transmission, particularly after rare horizontal transfer, as discussed for *Drosophila* [34,65,66].

Kellner and Dettner [67] noted that pederin is synthesized in about 90% of the females, and can be transferred to their offspring. The discovery of the pederin biosynthetic gene cluster led to the finding that the endosymbiotic Gram-negative bacteria, identified as closely related to *Pseudomonas aeruginosa*, were the producers of these compounds [68,69]. Kador et al. [41] found that *Pseudomonas*-like endosymbionts are located inside a structure of the female genitalia of *P. riparius,* based on FISH investigations. The *Pseudomonas*-like endosymbionts distributed in the female genitalia of *Paederus* species produce pederin as a defensive compound against insect and arachnid predators, and this does not apparently decrease the fitness of their hosts [70]. Maleki-Ravasan et al. [24] reported that the coinfection rates of both *Pseudomonas*-like endosymbionts and *Wolbachia* were 70.59% in females and 17.57% in males. Perhaps *Wolbachia* and *Pseudomonas* may interact with each other and with their *Paederus* beetles. It is unclear whether the *Pseudomonas* regulates the population of *Wolbachia* via pederin or not. Hence, the co-occurrence of *Wolbachia* and *Pseudomonas* in rove beetles may imply that *Wolbachia* is adapted to cope with adverse conditions triggered by *Pseudomonas* [71]. The nature of such potential interactions needs further investigation, and the effect of *Wolbachia* on reproduction in rove beetles also needs to be examined. 

## 5. Conclusions

This study demonstrated that *Wolbachia* from *P. fuscipes* belonged to supergroup B, based on an analysis of MLST genotyping. The infection density in adults was higher than in any other life stage, and the reproductive tissues in adults had the highest infection densities, with similar densities between the sexes. These findings provide a starting point for understanding *Wolbachia* infection dynamics in *P. fuscipes* and interactions with other components of the microbiota, and could be a potential area for future research.

## Figures and Tables

**Table 1:** Primer sequences and amplicon lengths of PCR products of target genes.

Gene/Region	Primers	Sequence (5′–3′)	Amplicon Length	Annealing Temperature	Reference
16S rRNA	16S_F 16S_R	TTGTAGCCTGCTATGGTATAACT GAATAGGTATGATTTTCATGT	870 bp	55 °C	[43]
*gatB*	gatB_F1 gatB_R1	GAKTTAAAYCGYGCAGGBGTT TGGYAAYTCRGGYAAAGATGA	471 bp	54 °C	[46]
*coxA*	coxA_F1 coxA_R1	TTGGRGCRATYAACTTTATAG TCTAAAGACTTTKACRCCAGT	487 bp	54 °C	[46]
*hcpA*	coxA_F1 coxA_R1	GAAATARCAGTTGCTGCAAA GAAAGTYRAGCAAGYTCTG	515 bp	54 °C	[46]
*ftsZ*	ftsZ_F1 ftsZ_R1	ATYATGGARCATATAAARGATAGTCR AGYAATGGATTRGATAT	524 bp	54 °C	[46]
*fbpA*	fbpA_F1 fbpA_R1	GCTGCTCCRCTTGGYWTGAT CCRCCAGARAAAAYYACTATTC	509 bp	59 °C	[46]
*wsp*	wsp1_F1 wsp1_R1	TGGTATTGGTGTTGGTGCAG AACCGAAATAACGAGCTCCA	158 bp	50 °C	[53]
RPS3	RPS3_F RPS3_R	CCCAGATAATCATTATCG CAGATTGAATGTGTGACAC	191bp	50 °C	[54]

## Supplementary Materials

The following are available online at https://www.mdpi.com/2075-4450/11/9/625/s1, Table S1: Accession number of various host lines used in this study.
