# Supplementary material for: Phylogeny and Density Dynamics of Wolbachia Infection of the Health Pest Paederus fuscipes Curtis (Coleoptera: Staphylinidae)
Source: Insects. 2020 Sep 11;11(9):625. doi: 10.3390/insects11090625 (PMC7564247; doi:10.3390/insects11090625)
Supplement: Supplementary file 1 [file insects-11-00625-s001.pdf]

Table S1 Accession number of various host lines used in this study

Species	ST	*gatB*	*coxA*	*hcpA*	*ftsZ*	*fbpA*	Supergrou
Drosophila melanogaster	1	AE017196	AE017196	AE017196	AE017196	AE017196	A
Drosophila innubila	10	DQ842428	DQ842280	DQ842391	DQ842316	DQ842354	A
Drosophila recens	13	DQ842431	DQ842283	DQ842394	DQ842319	DQ842357	A
Drosophila simulans	14	LK055284	LK055284	LK055284	LK055284	LK055284	A
Nasonia longicornis	24	DQ842443	DQ842295	DQ842406	DQ842331	DQ842369	A
Muscidifurax uniraptor	23	DQ842441	DQ842293	DQ842404	DQ842329	DQ842367	A
Jamides alecto	38	EU127676	EU127568	EU127622	EU127731	EU127784	A
Ephestia kuehniella	19	DQ842438	DQ842290	DQ842401	DQ842326	DQ842364	A
Drosophila simulans	17	DQ842433	DQ842285	DQ842396	AY508998	DQ842359	A
Drosophila neotestacea	11	DQ842429	DQ842281	DQ842392	DQ842317	DQ842355	A
Drosophila orientacea	12	DQ842430	DQ842282	DQ842393	DQ842318	DQ842356	A
Polyrhachis vindex	51	EU127686	EU127578	EU127632	EU127741	EU127794	A
Agelenopsis longystila	66	-	-	-	-	-	A
Hylaeus variegatus	70	KP183317	KP183270	KP183323	KP183298	KP183278	A
Pheidole gatesi	60	EU127699	EU127591	EU127645	EU127754	EU127807	A
Megastigmus bipunctatus	53	KJ535731	KF531859	-	KJ535728	-	A
Nasonia giraulti	25	DQ842442	DQ842294	DQ842405	DQ842330	DQ842368	A
Drosophila bifasciata	34	DQ842427	DQ842279	DQ842390	DQ842315	DQ842353	A
Metapone madagascarica	48	EU127682	EU127574	EU127628	EU127737	EU127790	A
Agelenopsis aptera	65	-	-	-	-	-	A
Agelenopsis aptera	67	-	-	-	-	-	A
Agelenopsis longystila	75	-	-	-	-	-	A
Gryllus firmus	21	DQ842439	DQ842291	DQ842402	DQ842327	DQ842365	B
Acraea eponina	4	DQ842418	DQ842270	DQ842381	DQ842307	DQ842344	B
Horaga onyx	39	EU127708	EU127600	EU127654	EU127763	EU127816	B
Tetranychus pueraricola	365	JX094422	JX094418	JX094407	JX094404	JX094399	B
Nasonia vitripennis	26	DQ842445	DQ842297	DQ842408	DQ842333	DQ842371	B
Lycaeides idas	36	EU127706	EU127598	EU127652	EU127761	EU127814	B
Hylaeus styriacus	41	KP183316	KP183269	KP183322	KP183297	KP183277	B
Spodoptera exempta	222	JN656947	JN656945	JN656946	JN656944	JN656948	B
Armadillidium vulgare	6	DQ842420	DQ842272	DQ842383	DQ842309	DQ842346	B
Paederus fuscipes	540	MT892884	MT892881	MT892880	MT892882	MT892883	B
Acraea encedon	3	DQ842417	DQ842269	DQ842380	DQ842306	DQ842343	B
Brugia malayi	35	DQ842421	DQ842273	DQ842384	DQ842341	DQ842347	B
Chorthippus parallelus	62	JN698882	JN698878	JN698880	JN698881	JN698879	B
Chorthippus parallelus	64	JN698882	JN698878	JN698880	JN698881	JN698879	B

dr
